# Anelloviruses versus human immunity: how do we control these viruses?

**DOI:** 10.1093/femsre/fuae005

**Published:** 2024-02-09

**Authors:** Anne L Timmerman, Antonia L M Schönert, Lia van der Hoek

**Affiliations:** Department of Medical Microbiology and Infection Prevention, Laboratory of Experimental Virology, Amsterdam UMC, University of Amsterdam, Meibergdreef 9, 1105 AZ, Amsterdam, the Netherlands; Amsterdam institute for Infection and Immunity, Postbus 22660, 1100 DD, Amsterdam, the Netherlands; Department of Medical Microbiology and Infection Prevention, Laboratory of Experimental Virology, Amsterdam UMC, University of Amsterdam, Meibergdreef 9, 1105 AZ, Amsterdam, the Netherlands; Amsterdam institute for Infection and Immunity, Postbus 22660, 1100 DD, Amsterdam, the Netherlands; Department of Medical Microbiology and Infection Prevention, Laboratory of Experimental Virology, Amsterdam UMC, University of Amsterdam, Meibergdreef 9, 1105 AZ, Amsterdam, the Netherlands; Amsterdam institute for Infection and Immunity, Postbus 22660, 1100 DD, Amsterdam, the Netherlands

**Keywords:** anellovirus, torque teno virus, innate immunity, adaptive immunity, immune evasion, virus–host interactions

## Abstract

One continuous companion and one of the major players in the human blood virome are members of the *Anelloviridae* family. Anelloviruses are probably found in all humans, infection occurs early in life and the composition (anellome) is thought to remain stable and personal during adulthood. The stable anellome implies a great balance between the host immune system and the virus. However, the lack of a robust culturing system hampers direct investigation of interactions between virus and host cells. Other techniques, however, including next generation sequencing, AnelloScan-antibody tests, evolution selection pressure analysis, and virus protein structures, do provide new insights into the interactions between anelloviruses and the host immune system. This review aims at providing an overview of the current knowledge on the immune mechanisms acting on anelloviruses and the countering viral mechanisms allowing immune evasion.

## Teaser

Anelloviruses and their persistence in stable balance with the human immune system.

Abbreviations APOBEC: Apolipoprotein B mRNA editing enzyme, catalytic polypeptide-like
C: cytosine
HIV: Human Immunodeficiency Virus
IFN: Interferon
IκB: Inhibitors-κB
IKK: Inhibitors-κB kinases
IL: interleukin
ORF: open reading frame
miRNA: microRNA
NF-κB: nuclear factor kappa light chain enhancer of activated B cells
PhIp-Seq: phage immunoprecipitation sequencing assay
PWH: people with HIV-1
TAIP: TTV-derived apoptosis-inducing protein
TLR: Toll-Like receptor
TTMDV: Torque teno midi virus
TTMV: Torque teno mini virus
TTV: Torque teno virus
U: uracil

## Introduction

Making up over 70% of the healthy human blood virome, anelloviruses are considered to be the principal constituent of the human virome (Freer et al. 2018, Liou et al. 2022). Dominating not only within an individual but also worldwide, they are hypothesized to be present in the entire human population (Niel et al. 2000, Virgin et al. 2009). To date, four human infecting genera have been discovered: *Alphatorquevirus* (Torque teno virus; TTV), *Betatorquevirus* (Torque teno mini virus; TTMV), *Gammatorquevirus* (Torque teno midi virus; TTMDV), and the recently discovered fourth *Hetorquevirus* (Torque teno hominid virus) (Varsani et al. 2021). Information on Hetorqueviruses remains to be explored. The composition of anelloviruses infecting an individual is referred to as the anellome (Kaczorowska and van der Hoek 2020). The composition of such anellome is highly individualized and differs not only in the genera present but also in the corresponding concentrations of anelloviruses (Kaczorowska et al. 2022b). After its initial establishment, which can be traced back to the first months of life, the anellome differs in its composition when compared to that of adults. A young infant’s anellome primarily harbors TTMVs and TTMDVs soon followed by the TTVs (Kaczorowska et al. 2022a). It is thought that only over time the anellome is shaped into its adult version, where the anellome is often dominated by TTVs (Kaczorowska et al. 2022b), and richer in men compared to women (Cebriá-Mendoza et al. 2023). As research on the period between the second year of life toward adulthood has not been conducted, the transition process is yet to be elucidated. Studies on the anellome of adults revealed that the composition in healthy people remains relatively stable over decades (Maggi et al. 2003, Kaczorowska et al. 2022b). Information on the dynamics or development of the anellome in elderly is not available, although it is known that anelloviruses increase with age (Cebriá-Mendoza et al. 2021). Despite increasing research efforts, the scientific community has been unable to relate anelloviruses to a disease, implying anelloviruses could be a commensal component of the human virome (Kaczorowska and van der Hoek 2020).

With its genome size ranging from only 2.0 to 3.9 kb, anelloviruses are small, single stranded, and circular DNA viruses of negative sense, that contain overlapping open reading frames (ORF) and an untranslated region (Miyata et al. 1999, Jones et al. 2005, Ninomiya et al. 2007). The protein encoded by the ORF1 gene is considered to be the greatest in size and encodes the viral capsid (Qiu et al. 2005, Sarker et al. 2016). The protein encoded by ORF2 has, in addition to being essential for virion assembly (Nawandar et al. 2022), a potential regulatory function, suggested to interfere with immune responses of the host (Zheng et al. 2008). TTV-derived apoptosis-inducing protein (TAIP), encoded within the same region as ORF2 but different reading frame, is thought to play a role in triggering apoptosis of the host cell (Kooistra et al. 2004, Hino and Prasetyo 2009, Prasetyo et al. 2009). The function of the proteins expressed by ORF3 and spliced mRNAs ORF1/1, ORF1/2, ORF2/2, and ORF2/3 are yet to be deciphered.

The lack of a functioning culturing method, a system with infectious anellovirus particles that can be passaged, (reviewed elsewhere Kaczorowska and van der Hoek 2020), has limited research on anelloviruses. Nevertheless, using clinical samples with varying immune statuses enables researchers to investigate the relationship anelloviruses may have with the human immune system. It is known that anelloviruses are susceptible to interferon (IFN). *In vitro* IFN-γ treatment of a TTV-transfected Hodgkin lymphoma cell line L428 and a TTV-transfected kidney cell line 293 revealed inhibited TTV DNA replication (de Villiers et al. 2009). In addition, people treated with IFN-α—due to an hepatitis C virus infection—generally showed decreasing TTV concentrations in blood (Chayama 1999, Toyoda et al. 1999, Nishizawa et al. 2000, Maggi et al. 2001). Whether this vulnerability to IFN is also applicable to TTMV and TTMDVs is thus far unknown. Next to that, several studies have investigated TTVs, or in some studies total anelloviruses, in organ immunosuppressed transplant recipients and found an inverse correlation between viral load and immune activity (Burra et al. 2008, Liu et al. 2021). In line with the abovementioned findings are the results presented by Shibayama et al. (2001) who revealed that individuals with AIDS display high prevalence and high concentrations of TTVs (Shibayama et al. 2001). Even though these findings show no causal relationships, and thus do not provide insights into specific mechanisms of virus–host interactions, these findings do indicate that the anellome must be under (some) control by immune surveillance.

In this review, we present an overview of the mechanisms discovered so far that that are likely involved in immune surveillance, as well as the mechanisms applied by anelloviruses to escape immune surveillance.

## The innate immune response

### APOBEC3 editing

One element of the innate immune defense, apolipoprotein B mRNA-editing catalytic polypeptide-like 3 (APOBEC3 or A3) exhibits antiviral properties due to the ability to deaminate cytosine (C) to uracil (U) in the viral genome (reviewed by Pecori et al. 2022). The introduction of mutations is thought to result in the production of dead-end genomes and thus impaired viral replication (Ball et al. 1999, Tsuge et al. 2010). The APOBEC3 family consists of seven proteins: A3A, A3B, A3C, A3D, A3F, A3G, and A3H, that differ based on their intracellular location, and the nucleotide context they prefer to deaminate (Pecori et al. 2022). APOBEC3-mediated editing has been found in the genome of many DNA and RNA viruses, such as herpesviruses, parvoviruses, hepatitis B virus, Human Immunodeficiency Virus (HIV), human papillomaviruses, and coronaviruses (Noguchi et al. 2007, Vartanian et al. 2008, Kukimoto et al. 2015, Warren et al. 2015, Ratcliff and Simmonds 2021, Kim et al. 2022).

First evidence for interactions between anelloviruses and APOBEC3 has already been suggested in 1999 by Ball et al. (1999), who found APOBEC3 editing in 1 out of 93 TTV reads using Sanger sequencing. These findings were consolidated in 2010 by Tsuge et al. (2010), who performed a study, also using Sanger sequencing, demonstrating that APOBEC3 editing could indeed be found in TTV genomes (Tsuge et al. 2010). Based on the specific nucleotide contexts in which the mutations were found, A3G and A3F were suggested as responsible candidates.

While Ball et al. (1999) and Tsuge et al. (2010) looked specifically at TTVs, a study of Poulain et al. (2020) predicted that APOBEC3 is most probably also acting on TTMVs and TTMDVs (Poulain et al. 2020), as A3-recognition motif depletion was found for all three genera. These predictions were recently confirmed by us. By next generation sequencing, we investigated hypermutations in 64 anellovirus isolates in healthy people, and revealed that all three human anellovirus genera are affected by C to U deamination, with some isolates more vulnerable to deamination than others (Timmerman et al. 2022). The U-containing genomes were packaged inside intact virus particles, which we know because the clinical materials were treated with DNAse prior to analysis. The fact that uracils were found in the genomes within intact virus particles suggests that editing must have occurred at the late stage of the replication cycle, since the mRNA encoding an intact viral capsid could not have been transcribed from edited DNA. According to the observed nucleotide context, we considered A3C, A3D, A3F, and A3H as potential candidates acting on the negative sense anellovirus genome (Timmerman et al. 2022). Of note, APOBEC3 activity did not seem to change an anellome, as isolates that were most affected by editing were rarely lost in their hosts during the > 30 years that we were able to follow chronic anellovirus infections in individuals.

The mutational activity of the APOBEC3 protein can be enhanced by IFN-α and IFN-γ (Peng et al. 2006, 2007, Noguchi et al. 2007, Koning et al. 2011). Interestingly, in the experiments performed by Tsuge et al. (2010) alanine aminotransferase flare ups found in hepatitis B patients, associated with increased IFN-γ levels, led to APOBEC3 editing in the hepatitis B virus, and—at the same time—TTV levels dropped below their detection limit (Tsuge et al. 2010). This suggests that the decrease in TTV levels, occurring during IFN-γ and IFN-α treatment, may potentially be due to APOBEC3 editing activation.

### TLR-9 activation

Another unknown is Toll-Like receptor-9’s (TLR-9) involvement in anellovirus recognition. TLR-9 is a Toll like receptor present mainly intracellularly in antigen presenting cells, recognizing unmethylated DNA. Although TLR9 triggering was found after exposure to TTV-DNA in mouse spleen cells (Rocchi et al. 2009), no studies have thus far confirmed TLR-9 involvement using whole viruses, e.g. anelloviruses obtained from clinical samples like blood or saliva.

## The adaptive immune response

### Antibody response

Antibodies, as part of the adaptive immune system, are an important immune defense mechanism against viral infections (Delves and Roitt 2000). They may, therefore, also play a crucial role in keeping anelloviruses in check. Indeed, antibodies against anelloviruses have been detected. In 2000, Itoh et al. (2000) detected that purified virions were generally immune complexed with IgG. Next, Ott et al. (2000) expressed an almost full length TTV genotype 1 ORF1 in *Escherichia coli* and performed a serological study in 70 individuals, including blood donors, hepatitis patients, and children. They found that 98.6% of the individuals were positive for antibodies against this protein, whereas only 76% of the individuals were PCR positive for TTV. In other studies a much lower prevalence of antibody reactivity was found. Tsuda et al. (1999) compared antibody prevalence in TTV genotype 1 PCR positive and negative individuals by testing reactivity to whole virions (Tsuda et al. 1999). Virions (10^5^ TTV DNA copies per ml) were obtained from a feces suspension, and immune precipitation found antibodies in 17% of the healthy individuals PCR positive for TTV, whereas 29% of the healthy individuals PCR negative for TTV show antibodies against the virus. This link between PCR-negativity and increased frequency of finding ORF1 antibodies was, however, not found in two other studies. For TTV genotype 1 (Handa et al. 2000) as well as for genotype 6 (Kakkola et al. 2008), IgG antibodies in serum were found at higher frequency in individuals that were PCR positive for TTV. It is noteworthy though that the TTV-PCRs used in the various studies can differ, either by being broad and detecting a large variety of TTV isolates, or narrow with detecting only one (TTV genotype 1) isolate. PCR-detection of a few or many TTV isolates in a sample may explain the discrepancies in the abovementioned studies. Moreover, anellovirus proteins are extremely diverse, species and genera share at most 69% and 44% of its ORF1 coding region, respectively (Varsani et al. 2021). Individuals can carry a wide variety of infecting anelloviruses (Kaczorowska et al. 2022b), and antibody cross-reactivity may or may be limited between species, genotypes or genera.

As antibody-mediated neutralization of a virus depends on the epitopes being targeted, unveiling the locations where antibodies bind is of interest. In 2022, Venkataraman et al. (2022) used AnelloScan, a phage immunoprecipitation-sequencing assay (PhIp-Seq) to analyze the binding ability and affinity of antibodies to TTV, as well as TTMV- and TTMDV-derived linear peptides from ORF1, ORF2 ORF3, and TAIP (Venkataraman et al. 2022). The assay was not limited to a single genotype of each genus, as no less than 800 different human anelloviruses isolates (326 TTVs, 357 TTMVs, and 146 TTMDVs) were tested in the AnelloScan. Their results indicate that the protein encoded by the ORF1 gene is the most common target of antibodies, followed by ORF2, whereas reactivity to ORF3 or TAIP was rarely found (Venkataraman et al. 2022). However, one must keep in mind that the 3D conformation of the phage-displayed peptide library might differ from the conformational shape of the full protein used in other studies. Kakkola et al. (2008) expressed six ORFs of TTV genotype 6 in eleven different combinations in bacterial cultures and insect cells as arginine-depleted constructs (Kakkola et al. 2008). They found, in contrast with the findings of Venkataraman et al. (2022), highest IgG reactivity against the protein encoded by ORF2 (28.6%) and ORF2/3 (23.8%), followed by ORF1 (14.3%), ORF2/2 (5.3%), and ORF1/1 (5.6%). No reactivity against ORF1/2 could be observed. The response towards ORF1 was strongest for the C-terminal part of the protein, and response against the N-terminus was lacking (Kakkola et al. 2008). The immunodominance of the C-terminal part of ORF1 was also found in the AnelloScan (Venkataraman et al. 2022).

The presence of antibodies targeting anelloviruses prompted a deeper investigation into the potential implications for anellovirus survival over time. Kakkola et al. (2008) were able to investigate antibody response of four individuals for several years (Kakkola et al. 2008). One of the individuals remained PCR positive for TTV genotype 6 for over 6 years, and was genotype 6-ORF1 IgG positive throughout the whole follow-up. A second individual became PCR-positive for genotype 6 during follow up, yet did not develop antibodies to the genotype 6-ORF1. Instead antibodies recognizing genotype 6-ORF2 protein were found. Interestingly, following this ORF2 antibody seroconversion, this individual became PCR-negative for genotype 6, and remained negative for 5 years until the end of the follow up. Venkataraman et al. (2022) also investigated antibodies targeting newly introduced anelloviruses in people receiving blood transfusions (Venkataraman et al. 2022). They found that antibodies specifically recognizing the newly introduced viruses were produced in only a minority of the recipients. However, 5 months post transfusion > 70% of the newly introduced isolates, whether with or without produced reactive antibodies, did not stabilize themselves in the anellome, suggesting that other immune pressure, e.g. T-cell immunity (see below), may also play a role in eradication of newly introduced anelloviruses.

### T-cell immunity

To date, no studies investigating T-cell immunity and anellovirus dynamics have been reported, however, by looking at immunodeficiencies with low CD4^+^ T cell counts some indirect evidence on T-cell-mediated control may be obtained. Shibayama et al. (2001) and Liu et al. (2021), looked at anelloviruses in treatment naïve people with HIV-1 (PWH) (Shibayama et al. 2001, Liu et al. 2021). Through comparison of the viral loads versus the CD4^+^ T cell count in PWH using PCRs and next-generation sequencing, they found an inverse correlation; individuals that had a lower CD4^+^ T cell count also displayed higher prevalence and higher concentrations of TTVs. Although these results do not provide insights into specific mechanisms, the results do indicate that the anellome must, at least in part, be controlled by T-cell immunity.

Another indirect T-cell study was done by Sospedra et al. (2005). They isolated and expanded CD4^+^ T cells from cerebrospinal fluid of a MS patient, and predicted that these cells recognize peptide motifs from the arginine-rich region of the TTV ORF1 protein (Sospedra et al. 2005). It must be mentioned though that arginine-rich motifs are found in various viruses and autoantigens, and the recognition, therefore, does not necessarily predict direct T-cell reactivity against anelloviruses specifically. Additionally, the peptides were recognized at moderate concentrations, which indicates low avidity of the T-cells, probably too low to be able to clear TTVs (Sospedra et al. 2005).

People who receive allogenic organ transplants are treated with immune suppressive medication, e.g. corticosteroids and/or anti-T-cell induction immunosuppressants. Focosi et al. (2015) showed decreased TTV load in kidney or liver transplant patients 7 days after anti-T-cell drug administration, by either administration of anti-thymocyte globulin or basiliximab (Focosi et al. 2015). This TTV drop also correlated with the reduction of number of lymphocytes in the first 15 days. Interestingly, the concentration of anelloviruses showed a 2-fold increase 30 days after transplantation, while the lymphocyte concentration remained low. Other medications known for T-cell inhibition are Calcineurin inhibitors (tacrolimus/cyclosporine) (Laupacis et al. 1982, Clipstone and Crabtree 1992). In liver transplant recipients, a rise in TTVs is observed 3 months after transplantation, and this rise in TTV concentrations is higher with increasing concentrations of calcineurin inhibitors (Focosi et al. 2014), indicating that T-cells play a role in the control of anelloviruses. However, so far no study has specifically searched for T-cells that become activated upon recognition of distinctive anellovirus antigens. Needless to say that further research is necessary to acquire (more) understanding of anellovirus immune surveillance via T-cells, but the large diversity in each individual anellome may hamper easy measuring of anellovirus-specific T-cell reactivity. An AnelloScan-like approach (with > 800 isolates covered) may also be needed here.

## Mechanisms of immune evasion

### Antibody immune evasion

Anelloviruses may escape host B-cell-mediated immunity by evolving at an antibody recognition site, to such an extent that an antibody can no longer bind (Liou et al. 2022). One potential site of ORF1 where this process may occur is the hypervariable P2 region, part of the spike domain that forms a crown-like structure in the capsid of anelloviruses (Liou et al. 2022, Butkovic et al. 2023). Nishizawa et al. (1999) investigated sequence divergence by sequencing TTV genotype 1 in sera of two patients over time (Nishizawa et al. 1999). They found that the majority of the amino acid changes occurred in this hypervariable P2 region. In addition, we have recently investigated the evolution of six individual anellovirus isolates, intrahost, over the span of 30 years and confirmed that the majority of ORF1 nucleotide substitutions that lead to a change of an amino acid (positive selection) occur in this hypervariable region (Kaczorowska et al. 2023). It is most likely that the strong pressure to mutate in the hypervariable P2 region in ORF1 is related to the benefit these amino acid changes provide in escaping antibody recognition. Kakkola et al. (2008), also found in one individual—followed over 6 years—that presence of antibodies recognizing genotype 6-ORF1 did not lead to clearance of the genotype 6 TTV (Kakkola et al. 2008). Although not tested, as no longitudinal genotype 6 sequencing was done, persistence in this individual may have been due to antibody escaping mutations in the hypervariable region.

### Suppression of NF-κB

A key pathway factor in innate and adaptive immunity is NF-κB (nuclear factor kappa light chain enhancer of activated B cells) signaling (Oeckinghaus and Ghosh 2009). By activation, NF-κB signaling regulates gene expression of various proinflammatory genes including inflammasome activation, and activation of innate immune cells. The gene family codes for five proteins: P50, P52, P65, RelB, and C-Rel (Hayden and Ghosh 2012). With respect to anelloviruses, Zheng et al. (2008) revealed, by using a western blot, that the protein encoded by ORF2 is able to suppress the proinflammatory NF-κB pathway by downregulating the translocation of the proteins p50 and p65 into the nucleus through inhibition of Inhibitors-κB α (IκBα) degradation (Zheng et al. 2008). Normally, the binding of IκBα to NF-κB in the cytosol prevents the nuclear translocation of NF-κB. Only after the phosphorylation of IκBα by IκBα kinases (IKK), leading to degradation of IκB, releases NF-κB, which allows translocation into the nucleus, and results in transcription of genes of proinflammatory genes (Oeckinghaus and Ghosh 2009). Therefore, by the ORF2 mediated inhibition of IKK, NF-κB translocation may be prevented, which subsequently inhibits the production of inflammatory responses threatening anellovirus survival (Zheng et al. 2008). As NF-κB can also function as a transcription factor of certain proinflammatory genes, ORF2 protein can, thus also indirectly cause downregulation of certain proinflammatory cytokines transcription, which was indeed seen for interleukin-6 (IL-6), interleukin-8 (IL-8), and COX-2 (Zheng et al. 2008).

### Anellovirus microRNA

MicroRNA (miRNAs) are small single stranded, noncoding RNAs, which can be encoded by the host, but also by the virus (Kincaid and Sullivan 2012). Viral miRNAs can generally play a role in the extension of the lifespan of infected cells, evasion of the immune system, and the regulation of lytic infection. Anelloviruses have been found to express both single-stranded and double-stranded miRNAs, of which at least one transcript was found in plasma exosomes, in as much as 98 out of 102 subjects (Vignolini et al. 2016). However, more research is needed to elucidate the potential immune modulating effects of anellovirus encoded miRNAs.

## Concluding remarks

Ever since their discovery in 1997 (Nishizawa et al. 1997), researchers have tried to shed light onto the interactions anelloviruses have with their human host. One main question remains, however, unanswered: How can viruses that are so omnipresent, with continuous virus presence in the circulation, be tolerated by their host? Other DNA viruses known for their chronic infection and lifelong presence in humans (e.g. varicella zoster virus, parvoviruses, and herpesviruses) turn to latency after acute infection, and only during reactivation these viruses can be detected in blood or other sites of the body. Anelloviruses behave differently with their continuous presence in blood and no clear latency. In theory, it could be that tolerance due to anellovirus-specific immune exhaustion plays a role. Immune tolerance is for instance seen in chronic infection of the liver with the hepatitis B virus, where high hepatitis B DNA and surface antigen (HBsAg) levels in blood results in tolerance due to (T-cell) immune exhaustion (Fisicaro et al. 2020).

Anelloviruses show a remarkable variability, probably caused by thousands or even millions of years of evolution. Not only does this variation prevent the development of simple immunological assays like antibody or antigen ELISA, the high diversity complicates a study on anellovirus–host interactions even further. We suggest that in future research, anelloviruses should not be approached as one virus, but several. Differences between subgenera and/or isolates were detected regarding increased anellovirus load in immunosuppressed individuals (Blatter et al. 2018), APOBEC3 susceptibility (Timmerman et al. 2022) and antibody reactivity (Venkataraman et al. 2022). It would be of interest to investigate whether subgenera, or isolates, are more immunogenic or tolerogenic compared to others. When doing such research it may even be needed to take the (genetic) make-up of the infected person into account, which might also dictate the make-up of someone’s anellome.

Together, the interactions between anelloviruses and the host immune system form an intricate balance that results in the persistence of an anellome in its host. Research is strongly limited by the lack of an adequate culturing system, or animal model and it forces researchers to use indirect measures to study virus–host interactions. There is a reasonable chance that, apart from hepatocytes, immune cells, including granulocytes, or stem cells may actually facilitate replication of the virus (Itoh et al. 2000, Luo et al. 2000, Okamoto et al. 2000, Yu et al. 2002, Hu et al. 2005, Kosulin et al. 2018; reviewed by Kaczorowska and van der Hoek 2020; reviewed by Taylor et al. 2022). Lymphocytes, although probably not the activated T-cells (Li et al. 2015), could also be susceptible for anellovirus infection (Yu et al. 2002, Leppik et al. 2007). Immune cells having a double role in anellovirus persistence—combatting the virus, and at the same time facilitating replication of the virus—may complicate studies on anellovirus–immune interactions even more.
